# Vaccine Production in Africa: A Feasible Business Model for Capacity Building and Sustainable New Vaccine Introduction

**DOI:** 10.3389/fpubh.2019.00056

**Published:** 2019-03-20

**Authors:** Geofrey Makenga, Stefano Bonoli, Emanuele Montomoli, Trent Carrier, Joachim Auerbach

**Affiliations:** ^1^Department of Molecular and Developmental Medicine, University of Siena, Siena, Italy; ^2^GSK Vaccines, Siena, Italy; ^3^Takeda vaccines, Inc., Chicago, IL, United States; ^4^GSK Vaccines Institute for Global Health, Siena, Italy

**Keywords:** vaccine production, technologies, regulatory capacity, Africa, business model

## Abstract

Africa has the highest incidence of mortality caused by infectious diseases, and remarkably does not have the capacity to manufacture vaccines that are essential to reduce mortality, improving life expectancy, and promoting economic growth. GAVI has significantly helped introduction of new vaccines in Africa but its sustainability is questionable, and new vaccines introduction post-graduation is rare. Conversely, Africa with its high population and economy growth is an increasing potential market for vaccines. This study aimed to investigate how investment for vaccine production in Africa could be triggered and in which way it could be affordable to most African governments or investors. The investigation was based on a literature review and supplemented by online questionnaires directed to global vaccine stakeholders, African governments and regulatory authorities. In-depth interviews with experts in manufacturing capacity implementation and regulatory capacity building in Africa complemented the study. We also developed business plan scenarios including facility costs calculations and a possible investment plan based on expert opinions and publicly available information from pertinent sources. We saw that, governments in Africa, show interest in vaccine production establishments but only with external support for investment. The common regulatory functionality gap was the quality control laboratories to test vaccine lots before regulatory release. The global vaccine stakeholders showed less preference in investment for vaccine production establishment in Africa. The diverse political ambitions among African governments make it difficult to predict and access the market, a prerequisite for competitive production. A feasible solution could be a small production facility that would use technologies with high yield at low costs of goods to cover the regional needs. A respective antigen production facility is estimated to cost USD 25 Million, an affordable dimension for investors or interested African governments. Attractiveness for the African market is deemed to be high when targeting diseases almost exclusively for Africa (e.g., malaria or invasive non-typhoidal salmonella). With a smart 5 years tangible implementation plan, marketing agreements within existing regional collaborations and with a strong political will, an African government alone or together with an investor could convince global vaccine stakeholders and investors to support.

## Introduction

### Current Challenges of Concern for Africa

According to the Global Burden of Disease Study, published in 2016 (1), generally, infectious diseases decreased during the previous decade as a leading cause of death, and much of this decrease was driven by reductions in large contributors to global mortality, including HIV/AIDS, malaria, tuberculosis, and diarrhoeal diseases. However, in African and Asian regions, infectious diseases are still the leading cause of death, especially in children <5 years of age (2). About 44.4% of children deaths in 2016 occurred in sub-Saharan Africa, and 24·8% in South Asia (3). Children in low and middle income countries (mostly in Africa and Asia) are at much higher risk, with a 34-fold higher death rate than children in high income countries (2). Moreover, about half of the burden is contributed by diseases that seem to be almost exclusively reserved in Africa, such as malaria and invasive non-typhoidal salmonella (iNTS), also referred to as diseases of poverty (4).

Vaccination is one of the most important medical practice ever introduced, it has been essential to reduce mortality, improve life expectancy and economic growth (5–7). Africa is lagging behind in realizing the opportunities of reducing burden of disease by vaccination. Thanks to the Global Alliance for Vaccines and Immunization (GAVI), vaccines are becoming widely introduced (8–10). However, the concern is how to sustain such vaccines and whether countries would afford new vaccine introduction after graduation from GAVI (11, 12). Even if economic cost-benefit evaluation is one of the criteria relevant for priority setting in health (13), decision on vaccine introduction for most African countries would likely depend on pure program cost (14, 15). The more vaccines introduced in a country, the more expensive is the country's vaccination programme in terms of vaccine procurement, cold chain capacity and programmatic logistics (16–19). For example, Ethiopia spends USD 150 Million annually on vaccine procurement of which USD 100 Million currently come from donors (20). Eventually, upon graduation, the Ethiopian government is expected to triple its budget allocated to vaccine importation to sustain its immunization programme (21). We have seen several countries, such as India, Nigeria, and Thailand, which are now focusing on local production possibilities rather than importation of new vaccines with support from GAVI.

The biological processes with their inherent difficulty to manufacture vaccine batches with consistent characteristics and quality have been a hurdle to capacity expansion. Therefore, transfer of technologies and production processes to facilities located in Africa is a particular challenge (22–24). Brazil and Cuba are good learning examples for vaccine production setup by public institutions (25–28), while India is an example for private manufacturers (26, 29). These countries committed to build or shape their own biopharmaceutical manufacturing capacity, initially focused on domestic needs and later expanded to supply international markets through the United Nations Children's Fund (UNICEF) and the Pan American Health Organization (PAHO). This became an important source of income despite of the technology transfer challenges (28, 30). The likelihood of success for Africa is favored by the predicted population and thus market growth. The population of the less developed countries was projected to increase to 2.9 Billion in 2100, and four African countries, Ethiopia, the Democratic Republic of the Congo, the United Republic of Tanzania and Uganda, will be among the twenty most populous countries in the world in 2100 (31). In addition, African economy is growing at a steady rate of 5–6%, led by the East African region (EAC) (20–26, 26–34).

### Vaccine Production Concepts for Africa

According to Plotkin et al. (24) there are particular challenges involved in vaccine production, including process development, process maintenance, lead time, production facilities, equipment, life cycle management, and product portfolio management. The authors emphasized the importance of a robust and stable manufacturing process and consistent component supplies over decades to ensure long life cycle of a vaccine in a market. These areas should be carefully considered when planning vaccine production investment in Africa. Failure to manage these risks can result in costly product recalls, suspensions from the market and penalties may be assessed if a manufacturer fails to fulfill supply agreements (24). In this view, the choice of production technologies has huge impact on success of vaccine production establishment especially in the current African environment. The technologies for vaccine production, mainly the expression systems, play an important role in the cost of production, in terms of process stability and maintenance, life cycle and lead-time. There are several expression platforms, each with its yield capacity, and some are complex to develop (35–37). They have an important impact on cost of goods (COGs) and thus on the price for an affordable vaccine (37). Gerke et al. (38) has shown, production process for outer membrane particles from genetically modified bacteria called Generalized Modules of Membrane Antigens (GMMA), where, even a relatively small production facility (e.g., a 500 L fermenter) could produce in excess of 100,000,000 doses of vaccine per year. Such a technology would be highly favorable for African vaccine production because of its simplicity and the low production costs at a high yield.

Production can be done in either traditional fermenters so called stainless steel fermenters or single-use systems (SUS) (39–41). The choice of SUS or stainless steel or a mixed approach would depend on specific needs and the production scale (42, 43), also considering regulatory requirements (37, 44, 45), commissioning (46, 47) and facility maintenance. This has to be planned from the early phase of facility construction. A weak National Regulatory Authority (NRA) would create serious difficulties for the national and global business of a vaccine manufacturer. Since 2010, after the World Health Organisation (WHO) assessed NRAs in Africa (48), there have been great development in African NRAs and some NRA became fully functional, though, usually for oversight of pharmaceuticals only and not yet for biopharmaceuticals like vaccines. For vaccines, they depend on WHO pre-qualification programme (WHO-PQ) or other competent NRA licensure before local marketing authorization (48). These aspects must be keenly thought through and covered in the implementation plan of facility establishment and maintenance in Africa.

## Methods

This was a review based on literature and pertinent websites, to investigate how investment for vaccine production in Africa could be triggered. The study was supplemented by online questionnaires developed in Google form and directed to specified organizations and African countries, aimed at determining their interest on vaccine manufacturing capacity implementation in Africa. The online questionnaires were structured differently according to the role of the respondent. The survey was conducted between June and September 2016 and the respondents included:

1. Local officials of sub Saharan African countries governmental institutions (public health and economy)
2. NRA officials of sub Saharan African countries and non-African developing countries with vaccine manufacturers
3. Members of Developing Countries Vaccine Manufacturers (DCVMs)
4. Global vaccine manufacturers / multinational companies (MNC)
5. Global vaccine stakeholders (defined as officials from advanced NRAs, Independent consultants who have worked on or pioneered vaccine production or operations in developing countries, and officials from non for profit organizations that do vaccine production and development for developing countries)
6. WHO-NRA capacity building officials

The online questionnaire was customized to each group, e.g., DCVMs' members received questions on investment costs, benefit of indigenous vaccine production, experience on possible challenges incurred during setup of their facilities, governmental incentives and regulatory capacity building. Information collected from online questionnaires was limited to accessibility of the respondents whose addresses were obtained from attendance list of international meetings, such as the African Vaccine Regulatory Forum (AVAREF), official website for a particular organization (MNC, DVCM, NRA, WHO) or through LinkedIn search. In addition, we conducted in-depth interviews with officials from the Bill and Merinda Gates Foundation (BMGF) and the African Vaccine Manufacturers Initiative (AVMI). This was to gather information on their view on investment in vaccine production, efforts already made and challenges for capacity building in Africa. Results were analyzed descriptively.

In collaboration with subject matter experts, we generated and qualified the business model on manufacturing capacity building in Africa. This included the development of high level planning scenarios for manufacturing capacity implementation, the feasibility evaluation, identification of related needs for regulatory capacity building in Africa and the description of the impact that enhanced manufacturing and regulatory capacity would have on new vaccine introduction in Africa and its sustainability.

The scientific committee of the University of Siena, Italy approved the study. Respondents were treated anonymously and were consented for their participation. Ethical review and approval was not required for this study in accordance with the local legislation and institutional guidelines.

## Results

### Results From Survey Questionnaires

In total 30 responses were collected from various stakeholders including African governments (4 out of 14 contacted), African NRAs (11 out of 22 contacted), WHO (2 responses), MNC (3 out of 5 contacted), DCVM (5 out of 30 contacted), and Global vaccine stakeholders (5 out of 6 contacted). We also had two in-depth interviews, one with three officials from the AVMI and the other one with an official from BMGF.

**Figure 1:**
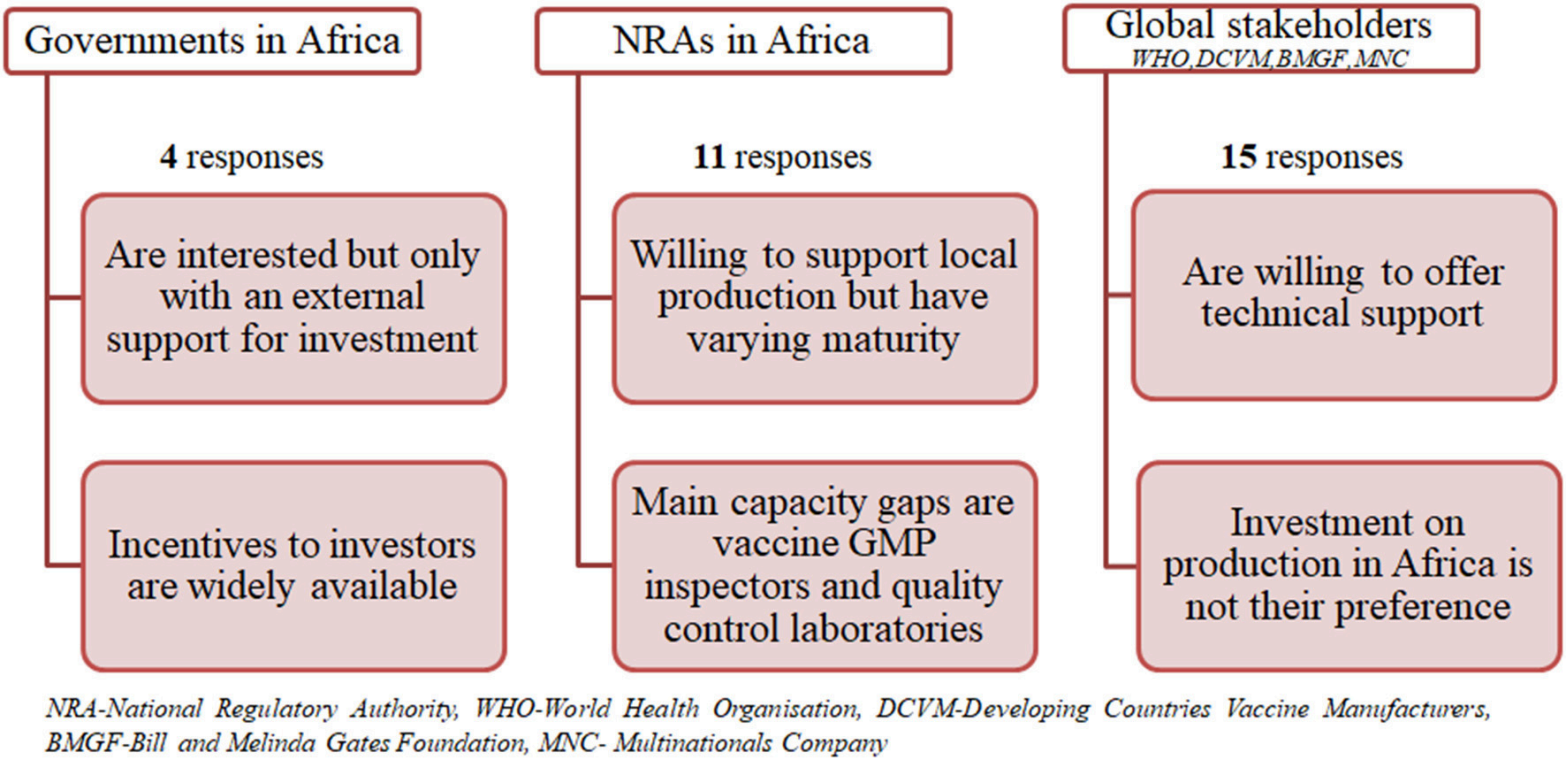
Summary of results from questionnaires.

Figure 1 shows the results summary from the questionnaires grouped into three categories namely governments in Africa, NRAs in Africa and global stakeholders (combining MNC, DVCMs, WHO, and BMGF).

### Governments Interest and Support

All responders were interested in the establishment of vaccine manufacturing capacity in their countries. However, they would do this only with an external financial and/or technical capacity building support. Thus, they are willing to support an investor at varying levels, such as land, tax incentives, infrastructure provision, and monetary support as a private public partnership. They are also willing to facilitate necessary extra capacity building in their NRA in a form of training or to support collaboration with competent authorities of other countries and the WHO. The latter would help them during an interim phase to cover all regulatory aspects around vaccine manufacturing facility establishment and at the initial stage of product life cycle. All responders expect access to a vaccine at an affordable price and establishment of employment for native experts.

### Regulatory Authority Capacity Gaps and Improvements

Capacity gaps vary from country to country; most NRAs would mainly need capacity building in relation to qualification of GMP inspectors, quality control laboratories for vaccine and technical expertise to perform lot release. They would require varying time for filling capacity gaps for indigenous production and supply, depending on availability of funding, from 1 to 5 years. The NRAs are willing to collaborate and rely on WHO and other competent NRAs from other countries to cover an interim period for regulatory need of vaccine manufacturing and batch release oversight. Some NRAs already perform batch release of all imported vaccines, e.g., Zimbabwe. With regard to global vaccine supply from an African country, some have already established regional regulatory collaboration, such as the EAC's Medicines Regulation Harmonization, making it easier for them to support each other in that regard.

### Developing Countries Vaccine Manufacturers' Perspective

Developing countries vaccine manufacturers are interested in seeing Africa developing their own manufacturing capacity; some would even expand their capacities to Africa in the future. However, the critical aspects for establishment of vaccine production capacity differ from country to country. Most of them are challenged by the access to expertise, source of raw materials, consumables, equipment, market access, country's import policy, and regulatory shortcomings in GMP inspection and long timelines for dossier review and approval. Other critical aspects included construction of facility, financial support, and acquisition of technology. Some government owned manufacturers would prefer such capacity to come from African government, as it is essential that a government will provide financial support for the project, construction, start-up of production, and commits to use the produced vaccine. The private manufacturers in India got supply of raw materials from the Indian government, which also provided incentives, such as sales tax, excise duty, and customs duty concessions as long as the unit was established in designated economic zones. DCVM acknowledged that indigenous vaccine production has significantly benefited their countries. The state owned manufacturers, such as Brazil base their success on their population (200 Million people with a 3 Million birth cohort) which is large enough for them to offer vaccine at a low price (similar to that of UNICEF) to their national immunization programmes. Hence their local production has provided security, avoiding shortage and strengthens their national technological capacity. Most private manufacturers in India have based their business on supplying vaccines to UNICEF and some claim to have reduced price to even half for indigenous purchase.

### Experts From Multinational Companies

As personal opinions, experts from multinational companies pointed out that feasibility for facility investment in Africa depends on the novel nature of the vaccine and the epidemiology of the disease, regulations etc. If a vaccine will provide high benefit in long-term for the majority of the African countries, there might be an opportunity to evaluate further the economic value of the vaccine and public health benefit via a business case. Some experts stressed the importance of research and development (R&D) costs in addition to facility investment costs for consideration when establishing vaccine prices. They also highlighted the importance of significant incentives like benefit of conducting business in a politically stable environment, ease of conducting business activities and regulatory navigation along the development and licensure roadmap, and tax incentives in the long term. Experts suggested that directing focus on Neglected Tropical Diseases (NTD) of particular interest for Africa would possibly lead to easier market development and eventually access to GAVI market.

### Global Vaccine Stakeholders

There are stakeholders who showed interest in establishment of vaccine manufacturing in Africa, reasoning that such capacity is needed in Africa, and would reduce cost of vaccines, help overcoming vaccine shortages, make countries better positioned to respond to outbreaks and would be a way for African countries to win their independence from the big pharmaceutical companies. It was suggested that, since no African country has enough inhabitants to justify the establishment of manufacturing capacities of significant size, African countries should identify a champion to build vaccine manufacturing capacities focusing on the needs of all African countries. In terms of support, most stakeholders were interested in investment at different levels. Based on their experience they believe that capital investment is likely substantial and therefore support from several NGOs, foundations, private companies will be crucial to facilitate vaccine manufacturing capacity implementation, including technology transfer and e.g., to establish advanced market commitments by securing financial resources. However, one of the experts was of the opinion that vaccines for Africa can be sourced more economically from countries like India, where a very robust vaccine industry exists.

### Role of WHO for Regulatory Capacity Building

The WHO is interested in establishment of vaccine manufacturing capacity in Africa and is willing to support the NRA where such capacity is being built. Basically, a country in which manufacturing capacity is being built should have appropriate technical skills, Quality Management System, market, and political commitment. Moreover, the ability to ensure adherence to international quality standards in a sustainable manner is a critical aspect for consideration. WHO's level of support is mostly technical, they have heavily supported regulatory system development in developing countries with vaccine manufacturing capacities, such as Brazil, India, Indonesia, and Thailand, and they did this through creating institutional development plans based on NRA assessment. In order to speed up regulatory capacity building in Africa, WHO officials suggest improvement on governance in countries and training of staff. There is doubt that global vaccine supply from a country in sub Saharan Africa is feasible from regulatory perspective in near or mid-term future. In any case it will require lots of funding and support by experts to become a reality.

### In-depth Interview With Experts

Experts from AVMI explained while there are several entities at various stages of vaccine production the current capacity of vaccine production in sub-Saharan Africa is mainly limited to Senegal's Institute Pasteur of Dakar (IPD), which produces yellow fever vaccine, the only WHO pre-qualified vaccine produced in Africa. Historically, most of the current vaccine facilities in Africa were government owned and competing priorities shifted the focus away from developing further capacity. However, the slow pace of progress in vaccine manufacture in Africa can be accelerated through political choice. Biovac is an example of how, with backing from the South African government and taking a reverse integration approach in building capacity across the value chain through partnering, globally recognized vaccine development and manufacturing capability can be established. It is critically important over the long term to reverse the current situation where <1% of vaccines used in Africa are made in Africa, leaving African countries vulnerable in emergency pandemic events. The same point was raised by the expert from BMGF who pointed out that commitment by African government has been a challenging as governments usually prefer cheaper vaccines from abroad over local ones, making it difficult for a manufacturer in Africa to have economies of scale to compete within the international vaccine market. It is envisaged that commercial agreement between African countries is necessary to ensure relevant local market size for return on investment. Nonetheless, governments' budget allocated to health is so limited and thus unlikely allows larger investment in vaccine manufacturing capacity building. Vaccine manufacturing remains complex and requires a highly integrated set of well-coordinated activities, including a highly skilled workforce, elaborate supply chain for many specialty reagents and consumables, exquisite quality control, and appropriately trained and staffed regulatory agencies. In view of current regulatory environment, it was recommended to first enhance pharmaceutical capacity and only in a second step (mid to long term) to build vaccine manufacturing capacity. BMGF is interested to accelerate availability of largest amount of high impact health products at best quality-price balance. Building manufacturing capacity for vaccines in Africa is not the most direct pathway to reach this objective. With regard to the technical or knowledge capacity building and the parallel development of the regulatory environment toward full functionality, BMGF collaborates strongly with the WHO.

### High Level Planning for a Vaccine Manufacturing Capacity Implementation in a Green Field in Africa

With the subsequent planning scenarios African governments and investors are supported to perform their own evaluation on investment feasibility, vaccine affordability, and market access possibilities, especially for vaccines targeted mainly or almost exclusively for Africa, such as malaria or invasive non-typhoidal salmonella.

#### Process Conceptual Plans

In general, a facility conceptual plan requires inputs from R&D and Marketing, especially technical information available at clinical phase 2 and sales forecast based on a clinical data driven initial marketing strategy. The following three production capacity building scenarios were developed based either on typical process-yield assumptions for a recombinant protein vaccine (49) or on published data for a GMMA based vaccine, all to be available in 2025, the earliest time point for availability of a validated facility, in case planning and implementation would start in 2018. The total yearly demand for an infant or toddler vaccine at a predicted birth cohort of 50 Million in Africa by 2025 (50), is 121 Million doses under the assumption of a 2-dose schedule, immunization coverage rate of 85% and the vaccine wastage of 30% (51, 52).

##### First scenario

As there is currently no concrete interest for investment, we arbitrarily selected a recombinant protein based vaccine as an example with the following assumptions: An investor in Europe or elsewhere has limited capacity (only one third) for producing and supplying a vaccine to Africa. According to the supply forecast, a yearly capacity to produce 121 Million vaccine doses will be needed by 2025. A fermenter will yield 75 mg/L of protein, if production is at a cycle time of 1 week. The resulting capacity will be ~21 Million doses per 1,000 L fermenter per year at a dose of 50 μg/0.5 ml. If an investor already produces 42 Million doses from an existing facility, which has two 1,000 L fermenters, the additional required capacity in the African country will be 80 Million doses from four production lines, each with a 1,000 L fermenter and respective purification capacity, assuming “like-for-like” technical transfer (this will create a total production capacity of 126 Million doses). The vaccine is adsorbed to aluminum hydroxide and filled into multidose vials. Respective formulation, fill and packaging capacities to produce 121 Million doses per year are required.

##### Second scenario

The second scenario is based on the first scenario, but here we use a single production line that results in 21 Million doses per year, sufficient to supply the EAC region. Alternatively, if production is targeted for a single country, such as Tanzania with estimated need of 7 Million doses a year, two other similar single antigen vaccines can be produced in campaign during the year.

**Figure 2:**
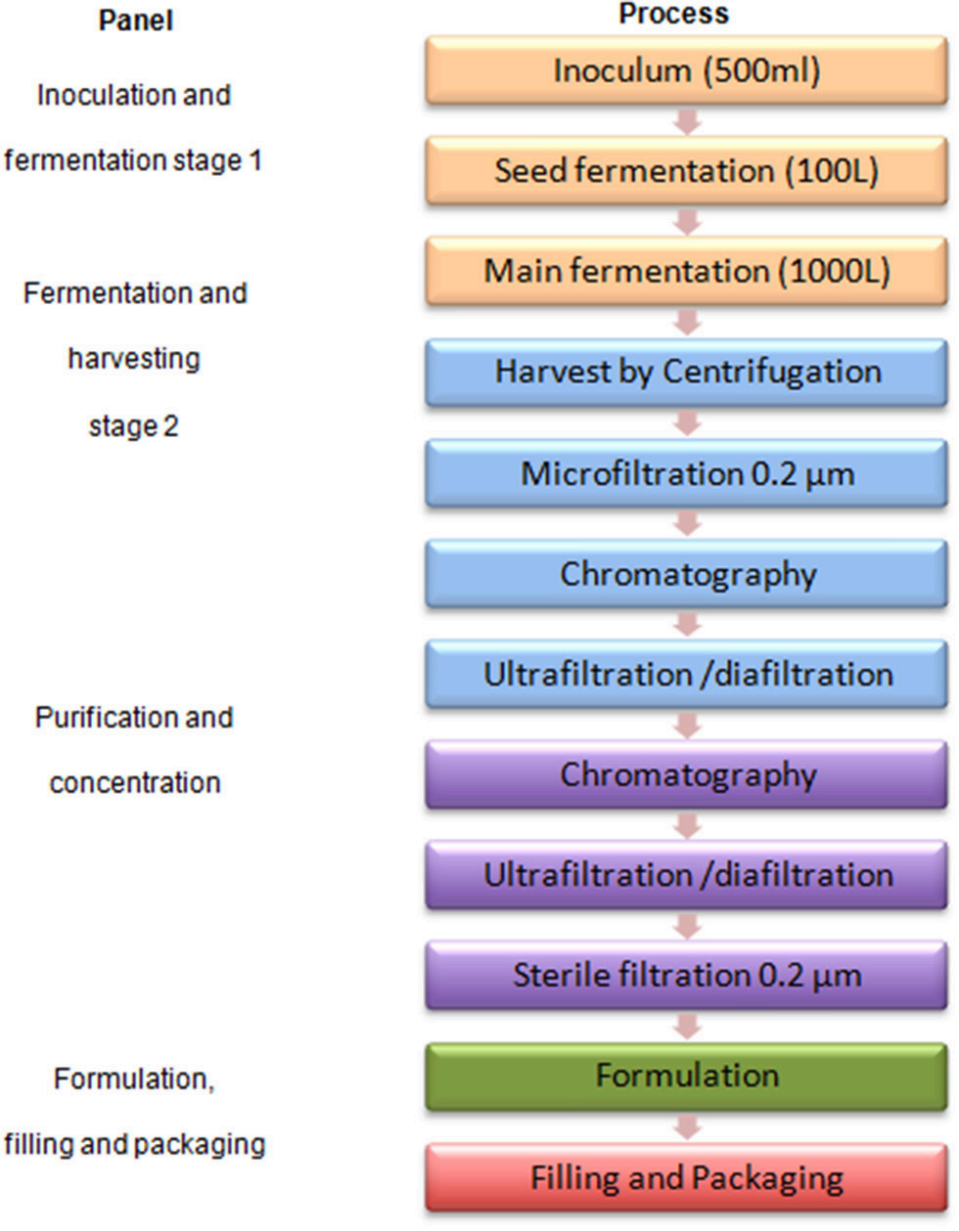
A typical protein antigen vaccine production process.

Figure 2 shows a typical protein antigen vaccine production process to feature first and second scenarios.

##### Third scenario

For vaccine production using GMMA technology as described by Gerke et al. (38), a relatively small production facility (e.g., a 500 L fermenter) could produce in excess of 100 Million doses of vaccine per year (38). The production time from setting up the inoculum for fermentation to final purified GMMA could be as short as 3 days per batch. Thus, depending on the size of human dose, with this kind of technology, just a single production line with a 500 L fermenter capacity would be almost enough to cover the whole African market need in 2025, assuming a 2-dose schedule infant vaccine.

**Figure 3:**
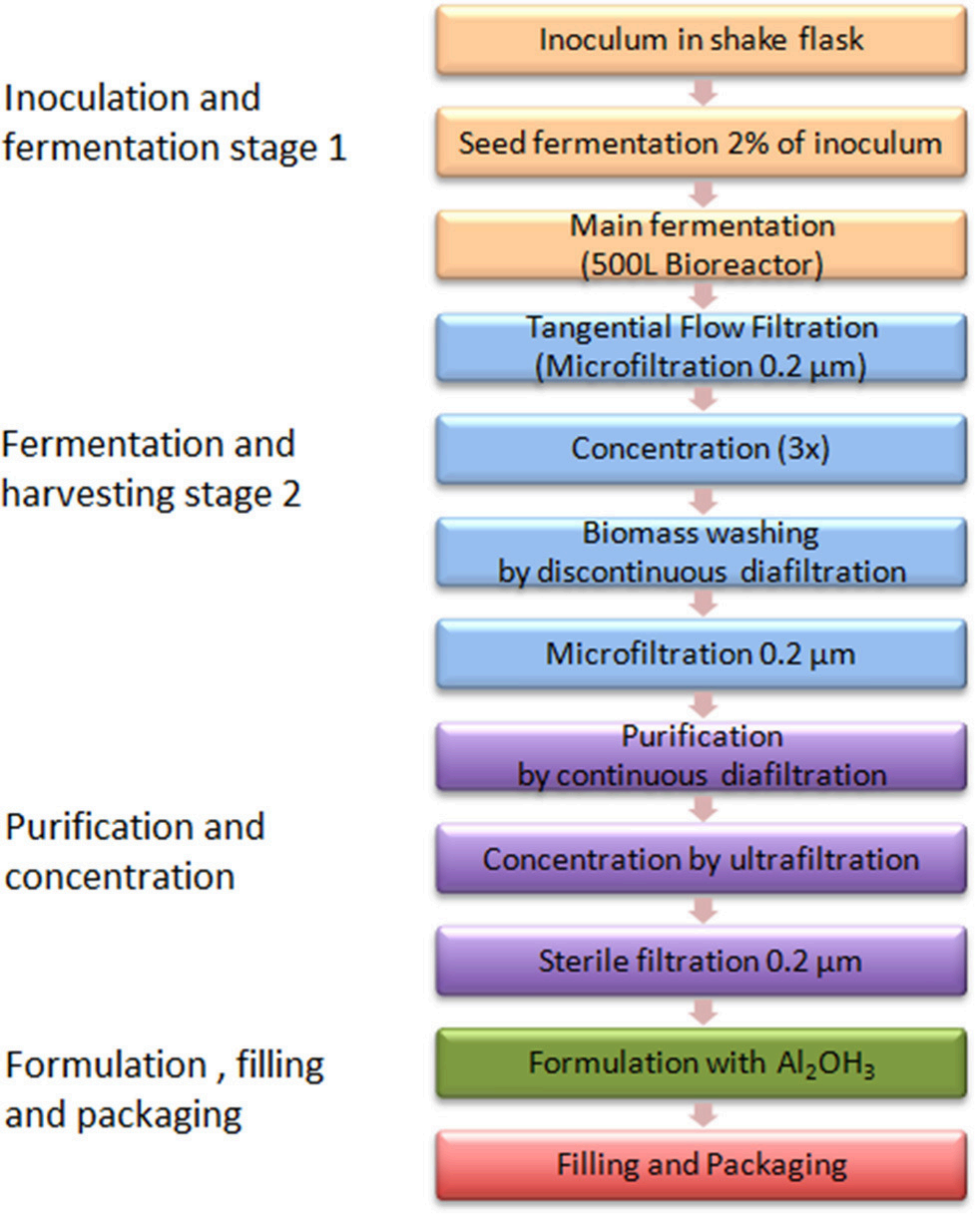
A GMMA based vaccine production process.

Figure 3 shows the GMMA based process flow diagram generated from process description by Gerke et al. (38).

#### Site Master Plan (SMP)

The SMP for all three scenarios follows the same concept and, in proportion, leads to the same cost estimates. Therefore, only the scenario for a new green-field facility, with a capacity to produce recombinant protein bulk for 84 Million doses and to formulate, fill and package 121 Million doses is planned. As described above, the fermentation technology platform is assumed to be well-known and for scenario 2 a flexible downstream capacity which has a capability to provide a range of recombinant protein vaccine products is required.

The size estimate assumptions for the SMP are developed in consideration of an “ideal site” with 75% of plant usage, providing 50% expansion capacity and production suites completely segregated with dedicated support areas and clean utilities. It is recommended that the facility location has to be well-characterized by considering aspects like land price, geographical conditions (flat, appropriate soil characteristics, easiness and security for foundation), the proximity to public water, energy supplies, sewer, waste disposal capabilities, transport connection, location in trade zones, topography of the site, type of neighbors and analysis of possible contrasts/synergies. The layout designs have to consider distances for personnel movements tailored to location of employees' parking spaces, guardhouse and canteen with respect to production buildings. They also have to describe relationships between buildings for main personnel flows, main material flow and utilities distribution requirements. This will then guide setup for construction phases.

#### Architectural Size Estimates

The bulk production building will accommodate four production lines with a separated purification suite. The formulation areas need to accommodate ~180 batches per year (44 cycles × 4 production lines). The filling area requires two vial filling lines using disposable flow-paths at a nominal capacity of 24,000 units/h to fill the 180 batches and the packaging area requires two lines, each with a nominal capacity of 12,000 units/h to finish the 121 Million doses. Furthermore, a warehouse, a QC and QA building with a 1:2 QA: QC ratio to accommodate laboratories and offices for 40 people and an animal house for 250 mice, 20 guinea pigs, 60 rabbits and a BSL2 testing room are required. The plan contains also a canteen with 100 seats and a building for administration that can accommodate 50 people. In addition, a guard house and parking lots are included. Space for central utilities, an electrical substation and a waste water treatment (WWT) plant to provide treatment for liquid effluents based on biological demand correspondent to sanitary streams of 1,000 people equivalent as worst case complete the dimension setting for the architectural planning.

The size of the production buildings was estimated based on a rule of thumb (53) as described in Table 1 below. For one Drug Substance (DS) production line, a process area of 3,500 sq. feet is estimated. Thus, assuming this process area is 17.5% of total area in a production building, the building for DS ptoduction with four lines will have a total process area of 14,000 sq. feet and hence, a total dimension of 80,000 sq. feet. For the second and third scenario, a single production line facility with process area of 3,500 sq. feet would require 20,000 sq. feet total building area. The areas of the other buildings are calculated following expert opinion and are shown in the Table 2.

**Table 1:** Rule of thumb for area distribution in a drug substance production building.

**Area**	**Size distribution**
Process support (labs, wash/prep, autoclave)	5 – 10%
Building circulation (corridors and airlocks)	8 – 17%
Personnel (lockers, offices, etc.)	5 – 15%
Material handling (storage)	5 – 15%
Mechanical (HVAC, utilities, electrical, chases)	35 – 50%
Process areas (fermentation, purification, buffer/media)	15 – 20%

**Table 2:** Size and cost estimates for vaccine manufacturing site scenarios.

**Components of a vaccine manufacturing site**	**First scenario**			**Second scenario**		**Third scenario**	
	**Cost per sq. foot (USD)**	**Area size (sq. feet)**	**Cost (USD) Million**	**Area size (sq. feet)**	**Cost (USD) Million**	**Area size (sq. feet)**	**Cost (USD) Million**
Master cell bank	1,000	1,000	1.00	250	0.25	250	0.25
Bulk production	1,250	80,000	100.00	20,000	25.00	20,000	25.00
Formulation	1,500	17,000	25.50	4,250	6.38	17,000	25.50
Filling	2,000	40,000	80.00	10,000	20.00	20,000	40.00
Packaging	1,000	40,000	40.00	10,000	10.00	20,000	20.00
QC&QA	500	30,000	15.00	7,500	3.75	30,000	15.00
Animal house	500	3,000	1.50	750	0.38	3,000	1.50
Warehouse	300	30,000	9.00	7,500	2.25	30,000	9.00
Administration	400	7,500	3.00	1,875	0.75	1,875	0.75
Guard house	300	500	0.15	125	0.04	125	0.04
Central Utilities	500	6,000	3.00	1,500	0.75	1,500	0.75
Canteen	400	3,000	1.20	750	0.30	750	0.30
Subtotal			**279.35**				
Estimate for 25% over capacity	804	64,500	52.00				
Estimate for preparation of 50% future extension	100	129,000	13.00				
Total			**344.12**		**69.84**		**138.09**

#### Fabrication, Construction, Schedule and Validation

All aspects for fabrication, construction and validation have to be properly addressed linking to regulatory requirements (53). Therefore, the investor should carefully select experienced engineering companies and consider using pre-fabricated modules for which qualification is in part already performed when they arrive. For construction one has to decide whether to use an onsite (stick built) approach, where assembly of walls, piping, steel, etc. has to be done on site, or an offsite (modular) approach, where the facility modules are built elsewhere and then shipped to site. The other alternative is a hybrid approach where the facility shell is stick built onsite while process equipment are built as skids at the equipment vender. This would allow for concurrent facility and equipment construction. However, the choice has to be made based on costs, validations and qualification steps to be followed for the different cases.

**Figure 4:**
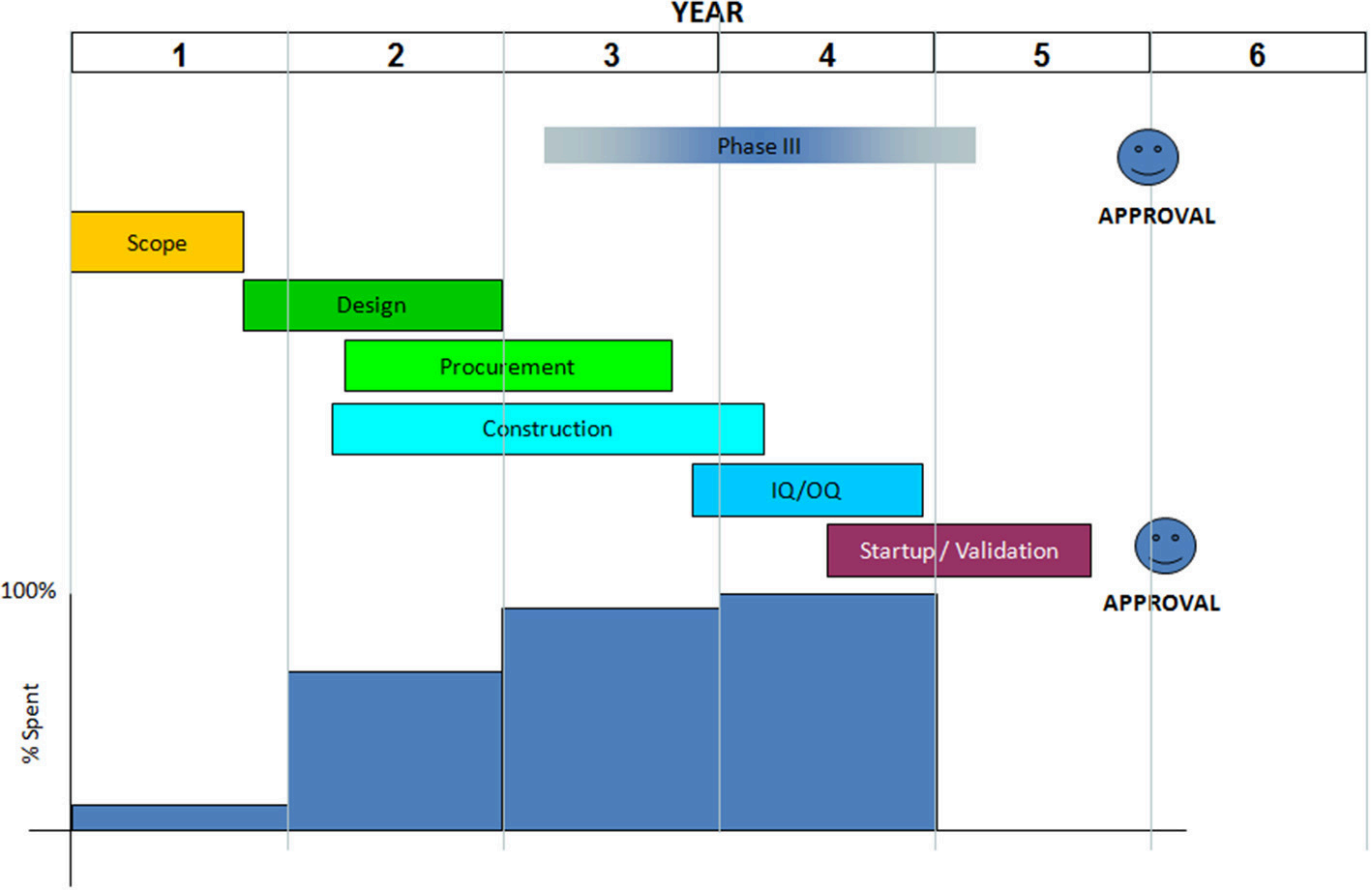
A typical vaccine manufacturing facility building schedule.

We recommend project schedule to be divided into scope, design, procurement, construction, Installation Qualification and Operation Qualification (IQ/OQ), start-up or Process Qualification (PQ), validation, and approval. The schedule should be timed in view of the date for expected launch of product produced in the new facility. Even though, the schedule duration is typically 5 years (see Figure 4), for the first vaccine manufacturing capacity implementation in Africa additional preparative time should be included in the plan. Extremely important will be to build sufficient scientific, technical and regulatory expertise, e.g., by sending scientists, technicians and regulators for training to sites/countries with process and regulatory expertise. For the execution, it is important to receive the building permission early and to plan and manage the construction phases in a way to minimize construction time. It could be better to use several expert companies with sufficient staff to work in parallel on sub-areas of the overall plan for the best and efficient project execution and meanwhile using collaborating institutions for global support on capacity building in human resources, improving NRAs and other aspects of technology transfer in each step.

#### Cost Estimates for Facility Construction

For Africa a modular type facility will be likely the best choice due to supposedly limited or lack of expertise in biopharmaceutical facility building. With the calculations following expert opinion based on a modular type facility, for which it can be assumed that costs are globally similar, we managed to come up with estimates for each of the three scenarios, which are detailed in Table 2. It should be taken into consideration that experts' facility cost estimation in the initial planning phase usually should be within an accuracy of ±30%.

The total cost for an ideal facility according to the first scenario would be about USD 344 Million. This could be reduced to about USD 280 Million not considering over capacity and future expansions. The second scenario with a cost of about USD 70 Million has a production capacity of 21 Million doses per year sufficient for regional supply (e.g., the six countries of EAC region). The third scenario costs about USD 138 Million and is able to supply either almost the whole African market with 1 single antigen vaccine or e.g., the EAC region with three different single antigen vaccines to cover the estimated demand in a region. However, only the concrete real scenario plan can provide accurate estimates on key components of a vaccine manufacturing site.

These cost estimates do not include the costs involved in start-up or Process Qualification (PQ) and validation activities. Usually at least 3–5 product batches will be produced during PQ and validation. There are significant costs involved but, ideally, these batches can later be sold. A full cost estimate for PQ and validation requires a detailed product knowledge that enables estimation of the COGs. Each facility project should include respective costs in the overall plan. In addition, facility-running costs should be calculated as they may impact the project as idle cost of the facility, once established and not used. In case of continuous use, these costs are absorbed into the COGs. The larger or complex the facility, the higher the running costs. However, we anticipate that labor costs in Africa would be low compared to Asia and Europe (54), even though at the beginning costs may even be higher, due to the need of high cost experts from high income countries (24). The involved cost risk i.e., long burden of start-up costs or idle costs (24) can be reduced by assuring full size market access in the initial facility establishment plan.

#### Product Cost Calculations and Marketing

Table 3 gives special considerations for vaccine cost calculations (53). Applying the respective criteria to the GMMA based technology (third scenario) this technology ensures a competitive vaccine price likely acceptable to UNICEF and affordable to African countries. The technology results in high yield and needs only few steps for purification, labor cost is greatly reduced (few people for few process steps), materials/consumables are highly reduced and due to the high yield there is room for scale increase in reasonable dimension. The other two scenarios will have higher manufacturing costs and are likely not as favorable as a GMMA based vaccine. Concrete estimates (24, 55) would be required to determine their competitiveness and acceptability to UNICEF and GAVI.

**Table 3:** Typical inputs for product cost calculations.

**Parameter**	**Drug substance cost (DS)**	**Drug product cost (DP)**	**Packaging cost**
General note	Main driver of DS costs are process yield and process scale	Largest contributor to DP costs is facility utilization	Largest contributor to packaging costs is the primary packaging material (e.g., vial, syringe)
Materials	Materials/consumables make up majority of variable costs for bulk production.	Standard materials/consumables make up small contributor. Specialty adjuvants will be incremental premiums.	Syringes can cost up to USD 1/unit Vials, stoppers, caps typically about USD 0.50/unit Lyophilized vaccines can require two vials (API and diluents) and process is costly.
Labor	Relatively small, overall contributor for automated facilities, large contributor for disposable equipment	Relatively small overall contributor	The packaging run size and configurations can become a significant cost determinant due to change over time
Overhead	Primarily fixed in nature but not large contributor due to productivity vaccine DS	Primarily fixed in nature (taxes, utilities, maintenance etc.) and can be large contributor.	
Scale	Scale increases can reduce costs per unit: (COGS2/COGS1) = (Scale 1/Scale 2)^(~0.4)^	Depreciation can be significant cost on a per dose basis (i.e., depreciation of USD 8–10 M over 10 M units = USD 1/unit)	

## Discussion

This study was a review based on literature and pertinent websites, to investigate how investment for vaccine production in Africa could be triggered. The study was supplemented by online questionnaires developed in Google form and directed to specified organizations and African countries, aimed at determining their interest on vaccine manufacturing capacity implementation in Africa. The response rate was limited, e.g., only four out of fourteen contacted African government officials responded. The low response rate had several causes, e.g., the email address was no longer valid as contacted persons had changed tasks or government officials were not comfortable to respond on behalf of their governments. However, in total 30 responses from various organizations were obtained and in-depth interviews with experts in the field complemented the information. We are therefore confident that the results are representative.

Governments in Africa show interest in vaccine production establishments, but only with external support for investment. However, investment for vaccine production establishment in Africa is currently not the preference of global vaccine stakeholders. Therefore, African governments should change their thinking, if they want to realize vaccine manufacturing implementation in their country. The key challenge is to ensure sufficient and assured market access for potential investors. Responses to the survey questionnaire showed that investors, including global vaccine manufacturers, are attracted more by market assurance than by provision of incentives (56, 57). Global stakeholders, such as GAVI and UNICEF can be involved to spearhead marketing aspects in the spirit of building capacity in Africa. Solutions in view of future challenges due to growth of African population and GAVI graduation will anyway be needed, and therefore, financial potential coming from projected growth of African economy could be directed into a program for self-sustainability with regard to vaccines. Interested governments can be approached for commitment to access an agreeable market through Africa's regional collaborations, such as EAC, SADC, ECOWAS, and AU, with consideration that a large enough population, like in India, China or Brazil, allows to grow due to an economy of scale. It is expected not to be easy getting neighbor countries to agree on common procurement of vaccines from an African manufacturer, but only this can guarantee a low affordable price and thus an investment with calculable risk. Disunity of African governments has delayed establishment and expansion of manufacturing capacity for both, vaccines and pharmaceuticals in Africa even though African economy and population growth were eminent and invited for investment.

Many African countries lack political will for concrete integration of pharmaceutical manufacturing development into economic development planning. However, e.g., Ethiopia has written and published a pharmaceutical manufacturing development plan (57) and the African Union Council (AUC) issued the Pharmaceutical Manufacturing Plan for Africa (PMPA) (58). Unfortunately, the plans do not show concrete political and or economical commitment to support implementation of pharmaceutical manufacturing capacity extension. The establishment of a vaccine policy by countries may assist in identifying how and when to consider local production. In particular, at the beginning, establishing local vaccine manufacturing is not necessarily cost-effective, but vaccines should not be seen purely as commodities. Factors, such as national health security should be considered as well (56). Committed governments, such as Cuba and Brazil have a requirement for public health delivery written in their constitutions (23, 25), despite their different historical backgrounds both countries have found a way to sustain their investment in public facilities and they have become role model in the PAHO region. African countries can learn from them.

Many African NRAs have developed capacities, including capacity for GMP inspection, for pharmaceuticals rather than vaccines due to globally driven traditional ways of vaccine procurement. If a country completely procures vaccines from UNICEF, it would not be cost effective and necessary for them to repeat tests already done by another competent authority. Therefore, such a country will likely remain dormant in NRA capacity development for vaccines unless a facility is established in that country. In Cuba, Brazil and India it was the development of vaccine production that pushed the governments to improve their NRAs. Improvement of regulatory oversight would also be needed in Africa, in particular as nowadays it is expected that a vaccine manufacturing facility implementation will be accompanied and approved by a fully functional NRA. Therefore, an interested African country should prepare early on by using available technical expertise provided by WHO and other stakeholders, such as AVMI for capacity building while a facility is being built in the country. For example, local training and research institutions can establish collaboration with abroad institutions (global vaccine stakeholders) and or companies for special vaccinology courses (59) and internship programmes aiming at building local capacity in terms of human resource that will work in both, NRAs and industries, with regard to vaccines.

Businesswise, investment in manufacturing capacity for a vaccine produced in a high yield technology, such as GMMA would favor quick return of investment at an affordable vaccine price for most African countries compared to production using a low yield technology. Building up a concrete plan within a specific country in Africa could even significantly lower the cost of investment, if e.g., available capacity from adjacent pharmaceutical industry with established QA/QC, formulation and fill and finish capacity could be built into the planning (brown field). Only the DS production facility for one antigen with a capacity for the whole African market would approximately cost USD 25 Million. Such a cost is affordable for most African governments as long as they include it in their economic plan and deliver within 5 years of implementation plan schedule. In addition, there is the opportunity that facility costs are lower in Africa as it has been shown that facility cost in Europe is even twice as high as in the Asian region (Russia, China, and India) (60–64). The brown field also offers an opportunity for existing private pharmaceutical companies to partner with an interested African government or a private investor for vaccine production tailored to market access.

Technical transfer can either be transfer of a licensed vaccine to extend capacity of a global manufacturer (first scenario) or an early transfer during development before or after proof of clinical concept. The first scenario would likely require an intense phase of vaccine specific knowledge and know how building of African scientists and technicians before or in parallel to the facility building and support by or transfer of key personal of the global manufacturer until robust process routine is achieved. The latter concept could be in collaboration with institutes like the GSK Vaccine Institute for Global Health (GVGH), the International Vaccine Institute (IVI), the Hilleman Laboratories, the Gates Medical Research Institute (GMRI), or other similar Institutes and could use a staggered approach: Building a GMP pilot facility that could be used for early clinical development (even as contract manufacturing site) and then extended to industrial or market scale (full capacity) for vaccine roll out according to demand forecast. This would also ease the technology transfer pathway and provide an opportunity to build staggered knowledge transfer. For a high yield process like GMMA a 250 L fermentation scale could be sufficient as industrial scale and then extended with 1 or 2 further production lines to cover the full African market. This would reduce investment risk and is an opportunity for any interested African country or its institutions to collaborate with global institutions focusing on vaccine development against neglected diseases of developing countries. International funds to support Phase 3 development of candidate vaccines against neglected diseases that have the potential to save many lives of African children will likely be required (65) in addition to a funding concept for the facility construction.

The Meningitis Vaccine Project (MVP) was a collaboration of the WHO (responsible for surveillance and vaccine introduction) and PATH (responsible for product development), who partnered with Serum Institute of India Private Ltd (SIIL) and public health officials across Africa to develop an affordable, tailor-made vaccine for use against meningitis A in sub-Saharan Africa (MenAfriVac). The project was set up after African leaders called for the development of a vaccine that would eliminate group A meningitis epidemics in Africa (66). The vaccine MenAfriVac was introduced via mass vaccination campaigns in 2010 and had a dramatic impact in reducing meningitis A epidemic. The project got funds from BMGF in 2001 (USD 70 M later added USD 17 M) to fight Meningitis in the meningitis Belt of Africa. In this collaboration SIIL supplied tetanus toxoid (TT) and Synco Bio Partners BV of Netherlands supplied Meningococcal A polysaccharide (MenA-PS). The FDA-CBER did the conjugation of MenA-PS with the carrier protein (TT) before transferring the production process to Serum Institute of India Limited (SIIL). The UK National Institute for Biological Standards and Control (NIBSC) did the testing of the vaccine batches produced by SIIL. The MVP shows how local institutions collaborated with international organization for capacity building on vaccine clinical development and disease surveillance. It also shows how international stakeholders could be involved for fast vaccine development and introduction, including capacity building and technical transfer. The success of the project mainly came from a strong political will geared by meningitis disease prevalence and mortality in that region. It was a good example showing that funds for clinical development of a vaccine that is almost exclusive for Africa can be obtained. MVP lowered investment costs for the producing industry (SIIL) and SIIL also benefited from the knowledge transfer on conjugation technology as that knowledge could be used to produce other vaccines. Africa definitely needs a partnership like for the MVP; this time as a long term development and/or manufacturing partner and not just as an end user. Such a collaboration project could build African capacity to overcome other problems, such as malaria, HIV, iNTS, etc.

We recommend that African countries should not start a very big complex project plan, which costs a lot of money and require sophisticated expertise and experience in vaccine manufacture. Instead they can make small projects targeting one antigen after another and grow over time. Preference should be given to already well-researched antigens or new antigens, which can be produced with simple, straightforward processes and for which there is no patent infringement. In addition, countries should quickly utilize their available high learning institutions and biotechnology research institutes to build sufficient indigenous technical expertise (human resources) required for vaccine production in collaboration with WHO, PATH, BMGF, and other institutes, which are dedicated to development of vaccines for Developing Countries, such as IVI, Hilleman Laboratories and GVGH. There would also be the opportunity to perform contract manufacturing during clinical development with the option to partner or acquire the project/vaccine at a later stage. This would allow a smooth and low risk phasing into realization of a sustainable vaccine manufacturing capacity and new vaccine introduction in Africa.

## Data Availability

The datasets generated on survey via google forms will be made available on request. Otherwise all relevant data is contained within the manuscript.

## Author Contributions

GM and JA: conceptualization. GM: writing original draft. GM and JA: data curation. GM, SB, TC, and JA: formal analysis. EM: funding acquisition. GM and JA: investigation. GM, SB, TC, and JA: methodology. SB, EM, TC, and JA: resources. JA: supervision. TC and JA: validation. GM: visualization. SB, EM, TC, JA: writing review and editing. EM: project administration.

### Conflict of Interest Statement

During the course of the study, GM spent 6 months as an intern at the GSK Vaccine Institute for Global Health, Siena, Italy in 2016. SB worked as a global operational engineer of GSK, Siena, Italy. TC worked with Takeda vaccines as a global operational engineer, and JA is the head of regulatory affairs at GSK Vaccine Institute for Global Health. TC is currently the Chief Executive Officer of CytoSen Therapeutics, Inc., North Carolina, USA. The remaining author declares that the research was conducted in the absence of any commercial or financial relationships that could be construed as a potential conflict of interest.
